# Role of Fracture Risk Assessment Tool and Bone Turnover Markers in Predicting All-Cause and Cardiovascular Mortality in Hemodialysis Patients

**DOI:** 10.3389/fmed.2022.891363

**Published:** 2022-04-07

**Authors:** Pei-Yu Wu, Szu-Chia Chen, Yi-Ching Lin, Po-Chih Chen, Wei-Shiuan Chung, Ya-Chin Huang, Ping-Hsun Wu, Yi-Chun Tsai, Jiun-Chi Huang, Yi-Wen Chiu, Jer-Ming Chang

**Affiliations:** ^1^Division of Nephrology, Department of Internal Medicine, Kaohsiung Medical University Hospital, Kaohsiung Medical University, Kaohsiung, Taiwan; ^2^Department of Internal Medicine, Kaohsiung Municipal Siaogang Hospital, Kaohsiung Medical University, Kaohsiung, Taiwan; ^3^Faculty of Medicine, College of Medicine, Kaohsiung Medical University, Kaohsiung, Taiwan; ^4^Department of Laboratory Medicine, Kaohsiung Medical University Hospital, Kaohsiung Medical University, Kaohsiung, Taiwan; ^5^Doctoral Degree Program of Toxicology, College of Pharmacy, Kaohsiung Medical University, Kaohsiung, Taiwan; ^6^Department of Radiology, Kaohsiung Medical University Hospital, Kaohsiung Medical University, Kaohsiung, Taiwan; ^7^Department of Radiology, Kaohsiung Municipal Siaogang Hospital, Kaohsiung Medical University, Kaohsiung, Taiwan; ^8^Department of Preventive Medicine, Kaohsiung Municipal Ta-Tung Hospital, Kaohsiung Medical University, Kaohsiung, Taiwan; ^9^Department of Occupational & Environmental Medicine, Kaohsiung Medical University Hospital, Kaohsiung Medical University, Kaohsiung, Taiwan

**Keywords:** Fracture Risk Assessment Tool, bone turnover markers, fracture, mortality, cardiovascular, hemodialysis

## Abstract

**Background:**

Fracture Risk Assessment Tool (FRAX) and bone turnover markers (BTMs) predict fractures in the general population. However, the role of FRAX and BTMs in predicting mortality remains uncertain in hemodialysis (HD) patients.

**Methods:**

One hundred and sixty-four HD patients stratified by low or high risk of 10-year fracture probability using FRAX. High risk of fracture was defined as 10-year probability of hip fracture ≥3% or major osteoporotic fracture ≥20%. The association of high risk of fracture and BTMs with all-cause mortality and cardiovascular (CV) mortality were evaluated using multivariate-adjusted Cox regression analysis.

**Results:**

Eighty-five (51.8%) patients were classified as high risk of fracture based on FRAX among 164 HD patients. During a mean follow-up period of 3.5 ± 1.0 years, there were 39 all-cause deaths and 23 CV deaths. In multivariate-adjusted Cox regression, high risk of fracture based on FRAX was independently associated with all-cause mortality [hazard ratio (HR): 2.493, 95% confidence interval (CI): 1.026–6.056, *p* = 0.044) but not with CV mortality (HR: 2.129, 95% CI: 0.677–6.700, *p* = 0.196). There were no associations between BTMs and mortality risk. Furthermore, lower geriatric nutritional risk index (GNRI) was significantly associated with increased CV mortality (HR: 0.888, 95% CI: 0.802–0.983, *p* = 0.022) after adjusting by confounding variables.

**Conclusion:**

High risk of fracture using FRAX was an independent predictor of all-cause mortality in patients undergoing HD. FRAX, rather than BTMs, has an important role of prognostic significance in HD patients.

## Introduction

Osteoporotic fractures can lead to physical dysfunction and decreased quality of life, and link to increased mortality and health cost in the general population (1–3). Accumulating evidence indicates that the fracture risk increases steadily with the loss of renal function in patients with chronic kidney disease (CKD) (4, 5), and the risk becomes four times higher in hemodialysis (HD) patients in comparison with healthy controls (6). Mineral and bone disorders, disturbed calcium and phosphate balance, secondary hyperparathyroidism, as well as vitamin D deficiency in chronic kidney disease contribute to the derangements in bone mineralization and turnover (7). Moreover, chronic diseases in CKD could aggravate frailty. Uremic toxin, inflammation, fluid overload, anemia, and malnutrition are involved in the process of muscle mass loss, cognitive impairment, and finally generate frailty in CKD (8, 9). Of note, frailty links to increased risk of fracture and mortality in dialysis-dependent patients (10, 11).

The Fracture Risk Assessment Tool (FRAX) is an online calculated tool, and is able to predict 10-year fracture risk based on clinical risk factors and bone mineral density (BMD) in the general population. Furthermore, FRAX helps doctors make treatment strategies for osteoporosis in clinical practice (12). Although the risk factors for major bone fractures in CKD are complex, FRAX has been demonstrated to stratify fracture risk in non-dialysis CKD patients and in HD patients (13–15). Bone turnover markers (BTMs) are series of biomarkers released during the process of bone remodeling. BTMs include markers of bone resorption and bone formation, and provide a non-invasive approach for studying bone turnover. Previous studies have reported a positive association between BTMs and fracture risk (16, 17). In patients with end-stage kidney disease (ESKD), BTMs were correlated with bone loss and low bone density (18, 19). However, the association between BTMs and fractures were conflicting in patients with CKD (19, 20).

Even though FRAX and BTMs might reflect the severity of low bone density and an increased risk of osteoporotic fractures, the associations of FRAX and BTMs with mortality risk remains unclear in patients with CKD or with ESKD. There are limited studies to examine their role in predicting mortality among this patient population. Hence, the aims of this study are to investigate the relationship between FRAX and BTMs, and further to evaluate the role of FRAX and BTMs in association with all-cause mortality and cardiovascular (CV) mortality in HD patients.

## Methods

### Study Participants

From March 2017 to December 2017, this interventional cohort study recruited 178 patients on thrice-weekly HD >3 months at the outpatient HD center of a regional hospital in Taiwan. All patients were ≥20 years of age and their each HD treatment lasted for 3.5–4.5 h based on the Kidney Disease Outcomes Quality Initiative clinical practice guideline (21). Patients who refused to undergo dual energy x-ray absorptiometry (DXA) scan (*n* = 6), patients with bilateral below knee amputation (*n* = 3), and those who were hospitalized 4 weeks prior the study enrollment (*n* = 5) were excluded from the study. Finally, a total of 164 maintenance HD patients (mean age 60.1 ± 10.6 years, 54.9% men) were included. The ethics review committee and Institutional Review Board of Kaohsiung Medical University Hospital approved the study protocol (KMUHIRB-F(I)-20150074). All patients provided their written informed consent.

### Demographic, Medical and Biochemical Information

Through the interviews and electronic medical records, the information of patients' demographic characteristics and their medical history were obtained. Fasting blood sample was collected for measurement of biochemical markers using an automated chemistry analyzer TBA-c16000 (Toshiba, Tokyo, Japan). The intact parathyroid hormone (PTH) assay was performed using the analyzer Immulite 2000 (Siemens Healthcare Diagnostics, Munich, Germany). Serum 25-hydroxyvitamin D concentration was analyzed using chemiluminescent microparticle immunoassay on an automated Abbott Architect i2000 (Abbott Laboratories, IL, USA). The geriatric nutritional risk index (GNRI), reliable indicator for nutritional status in maintenance HD patients (22), was calculated as GNRI = [14.89 × albumin (g/dl)] + [41.7 × (body weight/ideal body weight)] (23). The ideal body weight was defined as the value calculated from the height and a body mass index of 22 (24). If the patient's body weight was greater than the ideal body weight, body weight/ideal body weight was set to 1 (25).

### BTMs

Serum procollagen type 1 amino-terminal propeptide (P1NP) and C-terminal cross-linking telopeptide of type I collagen (CTX) were analyzed by an automated Roche electrochemiluminescence system (E411, Roche Diagnostics, Mannheim, Germany). The Unicel DxI 800 immunoassay system (Beckman Coulter Inc., Brea, CA, USA) was used to measure bone-specific alkaline phosphatase (BALP). Serum dickkopf-related protein-1 (DKK1) and sclerostin levels were measured using commercially available enzyme-linked immunosorbent assay (EIAab Science Co.:DKK1; Biomedica: sclerostin), following the manufacturer's protocols.

### Cardiothoracic Ratio and Aortic Arch Calcification

All patients' chest X-rays were reviewed by an experienced radiologist for assessment of the cardiothoracic ratio and aortic arch calcification (AoAC). The cardiothoracic ratio was calculated based on the ratio of the transverse diameter of the cardiac shadow to the transverse diameter of the chest on patients' chest X-rays. AoAC was assessed using the scale proposed by Ogawa et al. (26). In brief, the number of section with calcification was counted from the divided 16 sections of the aortic arch on patients' chest X-rays.

### Assessment of Fracture Risk Using FRAX

To assess the risk of fractures, FRAX is able to provide the 10-year probability of a major osteoporotic fracture and hip fracture, respectively. FRAX assessment requires the information about age, gender, height, weight, previous fracture, family history of hip fracture, current smoking, use of glucocorticoids, history of rheumatoid arthritis and secondary osteoporosis, amount of daily alcohol consumption, and femoral neck BMD (http://www.shef.ac.uk/FRAX). BMD at femoral neck was measured using a DXA scanner (Hologic Inc., Bedford, MA, USA). High risk of fracture was defined as 10-year probability of hip fracture ≥3% or a major osteoporotic fracture ≥20% (27, 28).

### Outcomes of Interest

Two clinical outcomes, all-cause mortality and CV mortality were assessed. CV mortality was defined as fatal myocardial infarction, fatal ventricular arrhythmia, sudden cardiac death, and fatal stroke. Study patients were followed until death, and the remaining patients were followed until July 2021.

### Statistical Analysis

Descriptive statistics are presented as percentages, mean ± standard deviation, or median (25th−75th percentile) for the dialysis vintage, AoAC, P1NP, CTX, BALP, DKK1, sclerostin, intact PTH, and high-sensitivity C-reactive protein (hs-CRP). The study patients were stratified into two groups based on low or high risk of fracture assessed by using FRAX. Differences between two groups of patients were analyzed by the chi-square test for categorical variables, and by an independent *t*-test for continuous variables with approximately normal distribution, or by Mann-Whitney *U* test for continuous variables with skewed distribution. Survival curves for all-cause mortality and CV mortality were illustrated using the Kaplan-Meier method and compared between above-mentioned groups of patients by the log-rank test. The univariate and multivariate adjusted Cox regression analyses were used to identify the factors associated with all-cause and CV mortality. The continuous variables with a skewed distribution were log-transformed to attain normal distribution in the Cox regression analysis. Significant variables (*p* < 0.05) in the univariate analysis were selected into the multivariate Cox analysis to identify the factors associated with all-cause and CV mortality. A *p* < 0.05 was considered statistically significant. All statistical analyses were carried out using SPSS version 22.0 (SPSS Inc., Chicago, IL, USA) for Windows.

## Results

### Clinical Characteristics of Study Patients

A total of 164 HD patients were included in this study. Table 1 lists the study patients' baseline characteristics. The median value of 10-year probability of hip fracture and major osteoporotic fracture were 3.35% and 8.35%, respectively. Eighty-five (51.8%) patients were classified as high risk of fracture based on FRAX. Patients with high risk of fracture were more likely to be older, had lower body mass index, lower GNRI, higher AoAC, lower femoral neck BMD, lower femoral neck T-score, and higher level of hs-CRP, compared to those with low risk of fracture.

**Table 1:** Comparison of baseline characteristics between maintenance hemodialysis patients with low or high risk of fracture assessed using FRAX.

	**All patients** **(*n* = 164)**	**Low risk of fracture** **(*n* = 79)**	**High risk of fracture** **(*n* = 85)**	***p*** **-value**
Age (year)	60.1 ± 10.6	53.9 ± 9.4	65.9 ± 8.1	<0.001
Men, *n* (%)	90 (54.9)	44 (55.7)	46 (54.1)	0.839
Smoking, *n* (%)	24 (14.6)	8 (10.1)	16 (18.8)	0.115
Diabetes mellitus, *n* (%)	85 (51.8)	38 (48.1)	47 (55.3)	0.357
Coronary artery disease, *n* (%)	23 (14.0)	12 (15.2)	11 (12.9)	0.679
Stroke, *n* (%)	13 (7.9)	4 (5.1)	9 (10.6)	0.115
Malignancy, *n* (%)	23 (14.0)	11 (13.9)	12 (14.1)	0.972
Dialysis vintage (year)	6.9 (3.3–11.3)	6.6 (2.5–12.5)	7.3 (3.9–13.4)	0.294
Body mass index (kg/m^2^)	23.9 ± 3.7	24.7 ± 4.4	23.2 ± 3.4	0.019
GNRI	98.4 ± 4.5	99.5 ± 4.1	97.4 ± 4.6	0.002
Systolic BP (mmHg)	155.5 ± 24.8	154.4 ± 24.2	156.6 ± 25.5	0.569
Diastolic BP (mmHg)	81.4 ± 14.3	83.2 ± 13.7	19.6 ± 14.7	0.836
Cardiothoracic ratio (%)	50.3 ± 5.0	49.5 ± 5.2	51.0 ± 4.7	0.053
AoAC	4 (2–7)	2 (0–6)	4 (5–9)	<0.001
Bone turnover markers				
PINP (ng/ml)	466.9 (285.5–887.5)	486.6 (312.6–886.4)	413.4 (269.7–890.2)	0.377
CTX (ng/ml)	2.34 (1.39–3.62)	2.45 (1.58–3.59)	2.24 (1.31–3.72)	0.859
BALP (μg/L)	17.4 (13.0–29.6)	17.9 (14.6–27.6)	17.2 (11.5–30.1)	0.507
DKK1 (pg/ml)	516.1 (357.2–731.5)	487.6 (295.9–743.6)	529.7 (370.3–733.8)	0.181
Sclerostin (pmol/L)	133.3 (99.4–179.1)	133.3 (103.3–164.9)	133.2 (96.2–187.7)	0.297
Biochemistry data				
Hemoglobin (g/dl)	10.3 ± 1.3	10.4 ± 1.3	10.3 ± 1.2	0.613
Total cholesterol (mg/dl)	172.2 ± 42.6	174.5 ± 40.8	170.0 ± 44.3	0.502
Triglycerides (mg/dl)	145.8 ± 121.2	158.0 ± 145.6	134.4 ± 92.4	0.213
Total calcium (mg/dl)	9.22 ± 0.90	9.23 ± 0.96	9.21 ± 0.83	0.875
Phosphorous (mg/dl)	4.59 ± 1.62	4.69 ± 2.14	4.49 ± 0.91	0.427
Magnesium (mg/dl)	2.48 ± 0.32	2.46 ± 0.32	2.49 ± 0.33	0.500
25-hydroxy vitamin D (ng/ml)	27.6 ± 9.6	28.5 ± 10.8	36.7 ± 8.4	0.242
Intact PTH (pg/ml)	301.0 (159.4–507.7)	280.8 (128.8–495.9)	346.9 (185.0–528.5)	0.218
hs-CRP (mg/L)	0.18 (0.09–0.47)	0.18 (0.06–0.38)	0.22 (0.12–0.66)	0.024
Femoral neck BMD (g/cm^2^)	0.59 ± 0.13	0.68 ± 0.11	0.50 ± 0.07	<0.001
Femoral neck T-score	−2.4 ± 1.1	−1.5 ± 1.0	−3.0 ± 0.7	<0.001
10-year probability of major osteoporotic fracture (%)	8.35 (4.83–15.0)	4.8 (3.1–6.8)	15.0 (9.9–21.0)	<0.001
10-year probability of hip fracture (%)	3.35 (1.60–7.63)	1.5 (0.5–2.2)	7.2 (5.2–13.0)	<0.001

*GNRI, geriatric nutritional risk index; BP, blood pressure; AoAC, aortic arch calcification; P1NP, procollagen type 1 amino-terminal propeptide; CTX, C-terminal cross-linking telopeptide of type I collagen; BALP, bone-specific alkaline phosphatase; DKK1, Dickkopf-related protein 1; PTH, parathyroid hormone; hs-CRP, high sensitivity C-reaction protein; BMD, bone mineral density*.

### Relationship Between BTMs and Clinical Features in Maintenance HD Patients

**Figure 1:**
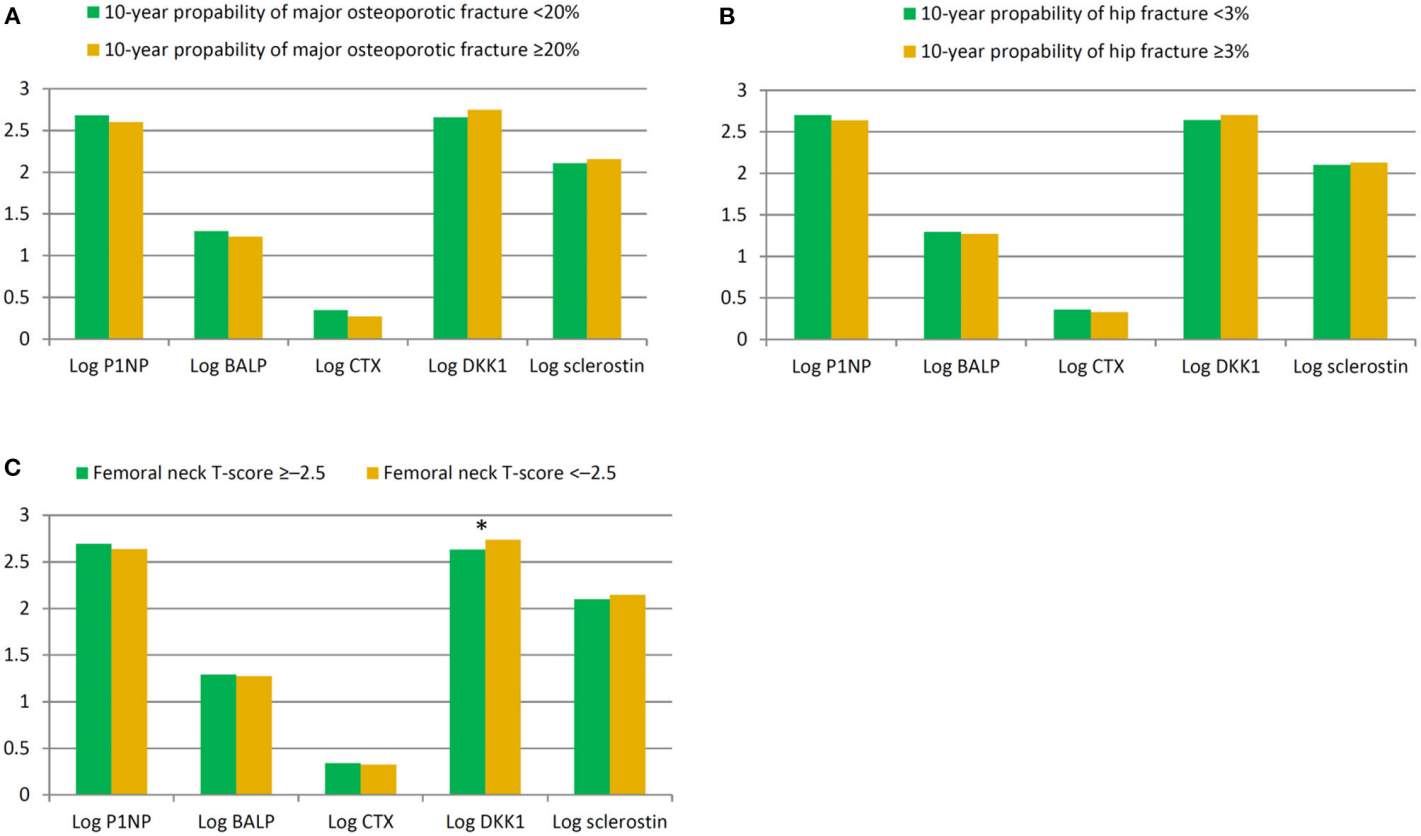
Comparison of bone turnover marks in patients stratified by 10-year probability of major osteoporotic fracture <20% or ≥20% **(A)**, 10-year probability of hip fracture <3% or ≥3% **(B)**, and femoral neck T-score >-2.5 or ≤ -2.5 **(C)**. *p = 0.004.

We further explored the relationships between BTMs and clinical characteristics in HD patients. As shown in Table 2, P1NP, BALP, CTX and sclerostin were not significantly correlated with any of study patients' clinical features. Of note, DKK1 was negatively correlated with hemoglobin, total calcium level and femoral neck T-score. Furthermore, we compared the differences of BTMs in patients with 10-year probability of major osteoporotic fracture <20% or ≥20%. Levels of P1NP, BALP, CTX were lower, while levels of DKK1 and sclerostin were higher in patients with 10-year probability of major osteoporotic fracture ≥20%, but these differences did not achieve statistically significance, as shown in Figure 1A. BTMs demonstrated in a similar fashion in patients with 10-year probability of hip fracture <3% or ≥3% (Figure 1B), and in those with femoral neck T-score ≥-2.5 or < -2.5 (Figure 1C). Specifically, level of DKK1 was significantly higher (*p* = 0.004) in patients with femoral neck T-score < -2.5 when compared to patients with femoral neck T-score ≥-2.5.

**Table 2:** Univariate correlations between bone turnover markers and clinical characteristics in maintenance hemodialysis patients.

	**PINP**		**BALP**		**CTX**		**DKK1**		**Sclerostin**	
	**rho**	***p***	**rho**	***p***	**rho**	***p***	**rho**	***p***	**rho**	***p***
Age (year)	0.009	0.913	0.048	0.562	−0.020	0.804	0.137	0.080	0.031	0.698
Dialysis vintage (year)	−0.017	0.824	0.012	0.875	0.019	0.807	−0.122	0.120	−0.096	0.221
Body mass index (kg/m^2^)	−0.101	0.199	−0.081	0.302	−0.081	0.300	−0.028	0.720	0.061	0.441
GNRI	−0.047	0.551	−0.042	0.592	−0.081	0.302	−0.092	0.242	0.086	0.272
Cardiothoracic ratio (%)	0.084	0.287	0.062	0.433	0.055	0.483	−0.012	0.884	−0.087	0.270
AoAC	−0.088	0.262	−0.049	0.530	−0.011	0.887	−0.014	0.854	0.056	0.479
Hemoglobin (g/dl)	−0.027	0.728	0.040	0.614	−0.046	0.560	−0.208	0.007	0.028	0.722
Total cholesterol (mg/dl)	0.007	0.927	0.025	0.750	0.010	0.899	−0.072	0.359	−0.059	0.454
Triglycerides (mg/dl)	−0.124	0.115	−0.098	0.213	−0.082	0.299	−0.105	0.181	0.021	0.787
Total calcium (mg/dl)	−0.085	0.277	−0.130	0.097	−0.004	0.960	−0.198	0.011	0.047	0.554
Phosphorous (mg/dl)	0.028	0.720	0.087	0.268	0.104	0.186	−0.031	0.692	0.020	0.795
25-hydroxy vitamin D (ng/ml)	0.061	0.439	−0.028	0.723	0.111	0.157	0.031	0.691	0.131	0.095
Intact PTH (pg/ml)	0.029	0.713	0.107	0.172	0.053	0.501	−0.050	0.525	−0.094	0.234
hs-CRP (mg/L)	0.005	0.953	−0.059	0.453	−0.043	0.583	−0.024	0.759	−0.079	0.312
Femoral neck BMD (g/cm^2^)	0.059	0.459	0.060	0.449	0.056	0.483	−0.104	0.190	−0.083	0.299
Femoral neck T-score	0.090	0.291	0.098	0.247	0.019	0.824	−0.168	0.047	−0.110	0.195
10-year probability of major osteoporotic fracture (%)	−0.037	0.640	−0.052	0.509	−0.063	0.422	0.100	0.205	0.084	0.283
10-year probability of hip fracture (%)	−0.053	0.502	−0.064	0.415	−0.060	0.449	0.122	0.121	0.086	0.288

*GNRI, geriatric nutritional risk index; AoAC, aortic arch calcification; P1NP, procollagen type 1 amino-terminal propeptide; CTX, C-terminal cross-linking telopeptide of type I collagen; BALP, bone-specific alkaline phosphatase; DKK1, Dickkopf-related protein 1; PTH, parathyroid hormone; hs-CRP, high sensitivity C-reaction protein; BMD, bone mineral density*.

### Factors Associated With All-Cause Mortality Using Cox Proportional Hazard Model

**Figure 2:**
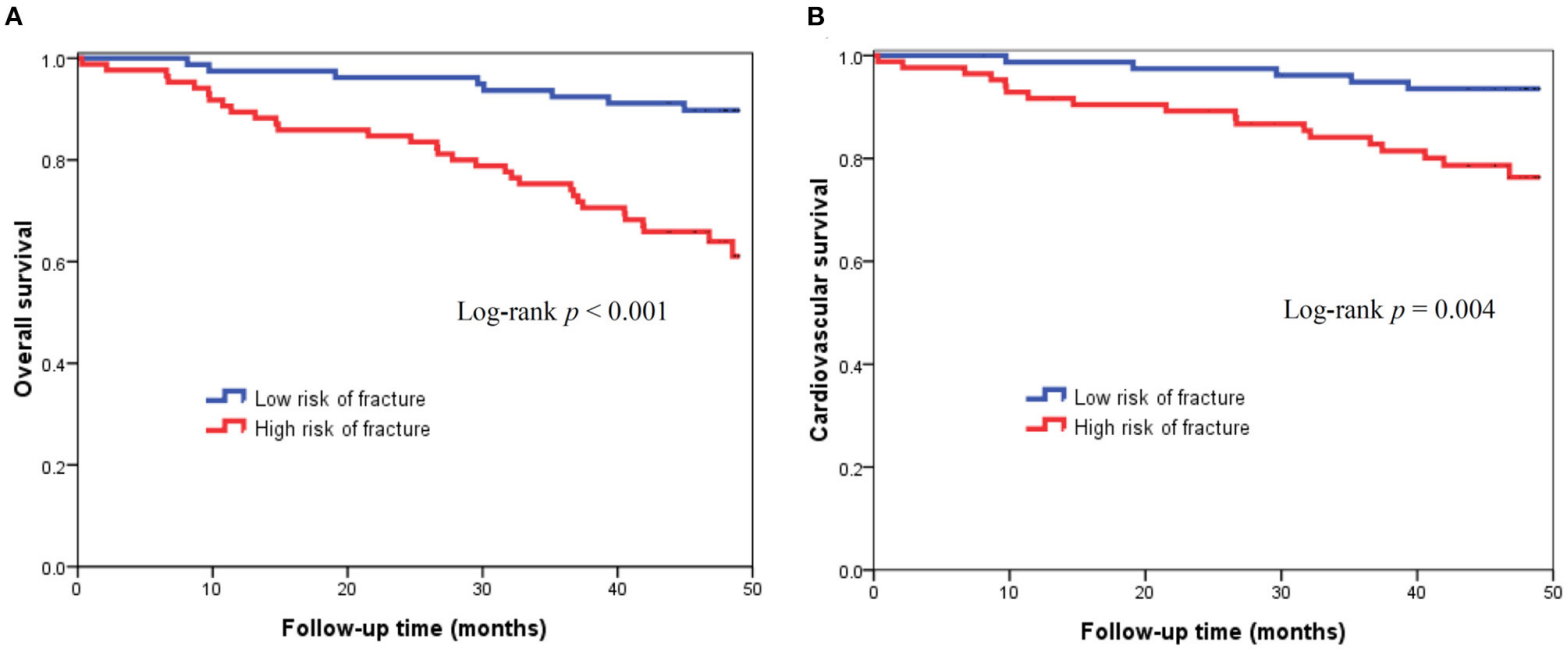
Kaplan-Meier curves of all-cause mortality (log-rank *p* < 0.001) **(A)** and cardiovascular mortality (log-rank *p* = 0.004) **(B)** according to low or high risk of fracture based on FRAX.

There were 39 (23.8%) deaths during a mean follow-up period of 3.5±1.0 years. The causes of mortality included CV death (*n* = 23), infectious disease or sepsis (*n* = 13), massive gastrointestinal bleeding (*n* = 2), and liver failure (*n* = 1). Figure 2A illustrates the Kaplan-Meier curves of survival according to low or high risk of fracture. Patients with high risk of fracture had a worse overall survival compare to those with low risk of fracture (Log-rank *p* < 0.001). In the univariate Cox proportional hazard analysis, high risk of fracture (hazard ratio [HR]: 4.281, 95% confidence interval [CI]: 1.966–9.322, *p* < 0.001), age ≥60 years, diabetes mellitus, coronary artery disease, higher hs-CRP, higher cardiothoracic ratio, higher AoAC, and lower GNRI were significantly associated with increased risk of all-cause mortality. In multivariate-adjusted Cox analysis, high risk of fracture (HR: 2.493, 95% CI: 1.026–6.056, *p* = 0.044) was independently associated with increased risk of all-cause mortality (Table 3).

**Table 3:** Factors associated with all-cause mortality in maintenance hemodialysis patients using Cox proportional hazards model.

	**Univariate**		**Multivariate**	
**Parameters**	**HR (95% CI)**	***p*** **-value**	**HR (95% CI)**	***p*** **-value**
High risk of fracture	4.281 (1.966–9.322)	<0.001	2.493 (1.026–6.056)	0.044
Age ≥ 60 years	4.320 (1.905–9.794)	<0.001	1.616 (0.635–4.115)	0.314
Men	1.431 (0.751–2.729)	0.276	–	–
Smoking	1.375 (0.606–3.117)	0.446	–	–
Diabetes mellitus	2.205 (1.117–4.355)	0.023	1.881 (0.933–3.792)	0.078
Coronary artery disease	2.395 (1.166–4.919)	0.017	2.022 (0.947–4.315)	0.069
Stroke	2.170 (0.909–5.183)	0.081	–	–
Malignancy	1.842 (0.846–4.009)	0.124	–	–
Dialysis vintage (per year)	1.004 (0.957–1.053)	0.882	–	–
BMI (per kg/m^2^)	1.006 (0.932–1.087)	0.871	–	–
GNRI (per unit)	0.896 (0.836–0.960)	0.002	0.928 (0.856–1.005)	0.067
Systolic BP (per mmHg)	1.006 (0.993–1.020)	0.359	–	–
Diastolic BP (per mmHg)	0.982 (0.960–1.003)	0.096	–	–
Cardiothoracic ratio (per %)	1.066 (1.000–1.137)	0.049	1.035 (0.968–1.107)	0.311
AoAC (per unit)	1.132 (1.057–1.213)	<0.001	1.048 (0.965–1.138)	0.268
Log P1NP (per log ng/ml)	0.501 (0.177–1.415)	0.192	–	–
Log CTX (per log ng/ml)	0.425 (0.132–1.367)	0.425	–	–
Log BALP (per log μg/L)	0.478 (0.132–1.730)	0.261	–	–
Log DKK1 (per log pg/ml)	1.487 (0.404–5.471)	0.551	–	–
Log sclerostin (per log pmol/L)	1.790 (0.291–11.005)	0.530	–	–
Hemoglobin (per g/dl)	0.925 (0.730–1.171)	0.517	–	–
Total Cholesterol (per mg/dl)	0.992 (0.984–1.000)	0.061	–	–
Log triglycerides (per log mg/dl)	0.436 (0.129–1.472)	0.181	–	–
Total calcium (per mg/dl)	0.887 (0.622–1.263)	0.505	–	–
Phosphorous (per mg/dl)	0.964 (0.771–1.207)	0.750	–	–
Magnesium (per mg/dl)	1.163 (0.457–2.961)	0.751	–	–
25-hydroxy vitamin D (per ng/ml)	0.986 (0.953–1.020)	0.410	–	–
Log intact PTH (per log pg/ml)	1.877 (0.768–4.583)	0.167	–	–
Ln hs-CRP (per Ln mg/L)	1.411 (1.111–1.792)	0.005	1.190 (0.946–1.498)	0.138
Femoral neck BMD (per g/cm^2^)	0.090 (0.006–1.424)	0.087	–	–
Femoral neck T-score (per unit)	0.754 (0.540–1.054)	0.099	–	–

*GNRI, geriatric nutritional risk index; BP, blood pressure; AoAC, aortic arch calcification; P1NP, procollagen type 1 amino-terminal propeptide; CTX, C-terminal cross-linking telopeptide of type I collagen; BALP, bone-specific alkaline phosphatase; DKK1, Dickkopf-related protein 1; PTH, parathyroid hormone; hs-CRP, high sensitivity C-reaction protein; BMD, bone mineral density*.

### Factors Associated With CV Mortality Using Cox Proportional Hazard Model

During the follow-up period, there were 23 CV deaths, including fatal myocardial infarction (*n* = 4), fatal ventricular arrhythmia (*n* = 2), sudden cardiac death (*n* = 13), and fatal stroke (*n* = 4). Figure 2B displays the Kaplan-Meier curves of CV survival according to low or high risk of fracture. Patients with high risk of fracture had a worse CV survival compare to those with low risk of fracture (Log-rank *p* = 0.004). In the univariate Cox analysis, high risk of fracture (HR: 3.935, 95% CI: 1.459–10.614, *p* = 0.007), age ≥60 years, diabetes mellitus, higher AoAC, and lower GNRI were significantly associated with increased risk of CV death. In multivariate-adjusted Cox regression analysis, diabetes mellitus (HR: 2.646, 95% CI: 1.020–6.869, *p* = 0.046) and lower GNRI (HR: 0.888, 95% CI: 0.802–0.983, *p* = 0.022) were independent predictors of CV mortality, but high risk of fracture was not associated with CV mortality after adjusting by confounding variables (Table 4).

**Table 4:** Factors associated with cardiovascular mortality in maintenance hemodialysis patients using Cox proportional hazards model.

	**Univariate**		**Multivariate**	
**Parameters**	**HR (95% CI)**	***p*** **-value**	**HR (95% CI)**	***p*** **-value**
High risk of fracture	3.935 (1.459–10.614)	0.007	2.129 (0.677–6.700)	0.196
Age ≥ 60 years	3.379 (1.253–9.112)	0.016	1.622 (0.533–4.942)	0.395
Men	1.383 (0.598–3.195)	0.449	–	–
Smoking	1.742 (0.647–4.697)	0.272	–	–
Diabetes mellitus	2.738 (1.079–6.948)	0.034	2.646 (1.020–6.869)	0.046
Coronary artery disease	2.421 (0.954–6.146)	0.063	–	–
Stroke	2.429 (0.836–7.231)	0.102	–	–
Malignancy	1.065 (0.316–3.587)	0.919	–	–
Dialysis vintage (per year)	1.017 (0.958–1.080)	0.573	–	–
BMI (per kg/m^2^)	0.995 (0.898–1.101)	0.995	–	–
GNRI (per unit)	0.881 (0.807–0.963)	0.005	0.888 (0.802–0.983)	0.022
Systolic BP (per mmHg)	1.007 (0.989–1.024)	0.460	–	–
Diastolic BP (per mmHg)	0.981 (0.954–1.010)	0.198	–	–
Cardiothoracic ratio (per %)	1.084 (0.997–1.179)	0.058	–	–
AoAC (per unit)	1.110 (1.013–1.216)	0.025	1.032 (0.923–1.153)	0.579
Log P1NP (per log ng/ml)	0.765 (0.193–3.034)	0.704	–	–
Log CTX (per log ng/ml)	0.539 (0.115–2.539)	0.435	–	–
Log BALP (per log μg/L)	1.080 (0.220–5.295)	0.924	–	–
Log DKK-1 (per log pg/ml)	3.443 (0.543–21.824)	0.189	–	–
Log sclerostin (per log pmol/L)	2.463 (0.222–27.303)	0.463	–	–
Hemoglobin (per g/dl)	0.854 (0.634–1.151)	0.300	–	–
Total Cholesterol (per mg/dl)	0.992 (0.982–1.003)	0.169	–	–
Log triglycerides (per log mg/dl)	0.719 (0.150–3.458)	0.681	–	–
Total calcium (per mg/dl)	0.661 (0.410–1.064)	0.088	–	–
Phosphorous (per mg/dl)	0.934 (0.671–1.298)	0.683	–	–
Magnesium (per mg/dl)	0.772 (0.199–2.987)	0.708	–	–
25-hydroxy vitamin D (per ng/ml)	0.994 (0.952–1.038)	0.790	–	–
Log intact PTH (per log pg/ml)	1.797 (0.567–5.691)	0.319	–	–
Ln hs-CRP (per Ln mg/L)	1.300 (0.949–1.782)	0.102	–	–
Femoral neck BMD (per g/cm^2^)	0.189 (0.006–5.995)	0.345	–	–
Femoral neck T-score (per unit)	0.857 (0.566–1.298)	0.467	–	–

*GNRI, geriatric nutritional risk index; BP, blood pressure; AoAC, aortic arch calcification; P1NP, procollagen type 1 amino-terminal propeptide; CTX, C-terminal cross-linking telopeptide of type I collagen; BALP, bone-specific alkaline phosphatase; DKK1, Dickkopf-related protein 1; PTH, parathyroid hormone; hs-CRP, high sensitivity C-reaction protein; BMD, bone mineral density*.

## Discussion

In this study, we examined the association among FRAX, BTMs and mortality risk in maintenance HD patients. Our results revealed that high risk of fracture using FRAX independently predicted all-cause mortality in patients on chronic HD. Besides, lower GNRI was also an important predictor of CV death in these patients. However, none of BTMs was associated with mortality risk.

Our study highlights the prognostic significance of FRAX in patients undergoing HD. FRAX has been extensively used to estimate 10-year fracture risk in the general population in various countries, and it helps decision-making process in management of osteoporosis (12, 29). However, the utility of FRAX has been questioned as chronic kidney disease-mineral and bone disorder (CKD-MBD) and multiple comorbidities may drastically affect turnover and remodeling of bones in patients with kidney disease. Over the last couple of years, mounting evidence has indicated that FRAX performs as well in patients with CKD or ESKD as in the general population. Whitlock et al. reported that FRAX was significantly discriminated risk of fractures in nondialysis CKD patients (13). In addition, FRAX and major osteoporotic fractures had a stronger relationship in CKD patients when compared with those with preserved kidney function (13). Przedlacki et al. analyzed 718 HD patients in a 2-year prospective study, which demonstrated that FRAX was the strongest independent risk factor for major bone fractures (14). Thus, FRAX is recommended in assessment of fracture risk and intervention in patients with CKD or ESKD in the updated consensus report (30).

Fracture events would increase the risk of subsequent unfavorable outcomes. In HD patients, the post-fracture risk of death was 3.7-fold higher in HD patients than those without fracture (31). However, few studies have elucidated the association between FRAX and mortality. In a retrospective study, Sezgin et al. showed that high fracture risk category of FRAX was associated with higher one-year mortality rate in 94 elderly patients (32). Hayashi et al. reported that higher major osteoporotic risk using FRAX was a predictor of death among incident HD patients in Japan (33). The findings in the present study were in line with these works. As for peritoneal dialysis patients, study in regard to investigation of the utility or the role of FRAX remains lacking. Furthermore, we also found that lower GNRI was also an independent predictor of CV mortality in this study. In the assessment of nutritional status in chronic HD patients, GNRI is an accurate indicator (34). We included 10-year probability of hip fracture in addition to major osteoporotic fracture using FRAX, and GNRI as a reliable nutritional marker, and CV death for analysis, which were not considered in the work by Hayashi et al. (33). Malnutrition, inflammation, multiple comorbidities, and nutrient loss from dialysis were risk factors of protein energy wasting and associated with frailty in patients with ESKD (8, 35). Frailty was prevalent in ESKD and significantly increased overall mortality risk regardless of age (10, 36). Recently, an association between a higher FRAX-score and frailty has been recognized (37). Overlapped risk factors for frailty and FRAX, including age, female sex, smoking, alcohol intake, multimorbidity and micronutrient deficits, might underlie the association between FRAX and increased mortality risk. Malnutrition was also the risk factor of frailty (38), and this might explain why a lower GNRI was associated with unfavorable outcomes in our study.

The regulation of bone formation and resorption involving in CKD-MBD is complex. Additionally, Wnt/β-catenin pathway increases osetoblastogenesis and decreases osetoclastogenesis, and it also plays an important role in bone remodeling process of CKD-MBD (39). P1NP and BALP are the markers of bone formation, whereas CTX, DKK1, and sclerostin are the markers of bone resorption, and DKK1 and sclerostin can inhibit Wnt/β-catenin pathway (19). The associations among BTMs, fracture, and BMD remain controversial in HD patients. A previous study found BTMs (P1NP, BALP, and CTX) were not associated with fracture, but were negatively associated with hip BMD in ESKD (19). However, Iimori et al. reported that BALP was associated with fracture in HD patients (40). Furthermore, the relationships between sclerostin, DKK1 and bone turnover were contradictory in patients with CKD (41, 42). In our study, only DKK1 was significantly higher in patients with femoral neck T-score < -2.5 and was consistent with a prior study in CKD (42). This result might be related to Wnt/β-catenin pathway inhibition. We also demonstrated that BTMs were not helpful in prediction of mortality in HD patients. Although certain studies indicated the link between serum sclerostin and adverse outcomes (43, 44), a meta-analysis and a recent prospective study showed that it was not associated with overall and CV mortality in HD patients (45, 46). The controversy in prognostic significance of BTMs in maintenance HD patients may be attributed to the differences in method of BTMs measurement, study heterogeneity, and different observation time.

There were several study limitations. First, this study enrolled HD patients in a single hospital with relatively small sample size. Second, we did not investigate all known BTMs and we checked BTMs only once at baseline. Since the BTMs are dynamic markers as changes in bone turnover and mineralization in CKD-MBD, it may be difficult to clearly address the association between BTMs and mortality risk. Third, the association between FRAX and fracture was not assessed because we cannot obtain the fracture events that were diagnosed in other hospitals. Finally, because age is an essential variable in the FRAX algorithm, a large-scale cohort study with age-matched patients is needed to better elucidate the role of FRAX in predicting mortality among chronic HD patients.

In conclusion, our study demonstrated that high risk of fracture using FRAX and lower GNRI independently predicted all-cause and CV mortality, respectively, in patients with HD. However, BTMs were not able to predict mortality risk. FRAX, rather than BTMs, has an important role of prognostic significance in maintenance HD patients.

## Data Availability Statement

The original contributions presented in the study are included in the article/supplementary material, further inquiries can be directed to the corresponding author.

## Ethics Statement

The studies involving human participants were reviewed and approved by the Institutional Review Board of Kaohsiung Medical University Hospital. The patients/participants provided their written informed consent to participate in this study.

## Author Contributions

J-CH: conception and design. P-YW and J-CH: writing–original draft. S-CC and J-CH: writing–review and editing and statistical analysis. P-YW, S-CC, Y-CL, P-CC, W-SC, P-HW, Y-CT, and J-CH: acquisition of data. S-CC, Y-CH, and J-CH: analysis of data. P-YW, S-CC, and J-CH: interpretation of data. Y-WC and J-MC: supervision or mentorship. All authors have read and agreed to the published version of the manuscript.

## Funding

The study was supported partially by a grant from the Ministry of Science and Technology, Taiwan (MOST 110-2622-H-037-001), and by a grant from Kaohsiung Municipal Siaogang Hospital (H-110-007), Kaohsiung Medical University, Kaohsiung, Taiwan.

## Conflict of Interest

The authors declare that the research was conducted in the absence of any commercial or financial relationships that could be construed as a potential conflict of interest.

## Publisher's Note

All claims expressed in this article are solely those of the authors and do not necessarily represent those of their affiliated organizations, or those of the publisher, the editors and the reviewers. Any product that may be evaluated in this article, or claim that may be made by its manufacturer, is not guaranteed or endorsed by the publisher.
